# Characterizing structural association alterations within brain networks in normal aging using Gaussian Bayesian networks

**DOI:** 10.3389/fncom.2014.00122

**Published:** 2014-09-30

**Authors:** Xiaojuan Guo, Yan Wang, Kewei Chen, Xia Wu, Jiacai Zhang, Ke Li, Zhen Jin, Li Yao

**Affiliations:** ^1^Information Processing Lab, College of Information Science and Technology, Beijing Normal UniversityBeijing, China; ^2^State Key Laboratory of Cognitive Neuroscience and Learning, Beijing Normal UniversityBeijing, China; ^3^Computational Image Analysis Lab, Banner Alzheimer's Institute, Banner HealthPhoenix, AZ, USA; ^4^Laboratory of Magnetic Resonance Imaging, Beijing 306 HospitalBeijing, China

**Keywords:** aging, Bayesian networks, structural association, gray matter volume, structural networks

## Abstract

Recent multivariate neuroimaging studies have revealed aging-related alterations in brain structural networks. However, the sensory/motor networks such as the auditory, visual and motor networks, have obtained much less attention in normal aging research. In this study, we used Gaussian Bayesian networks (BN), an approach investigating possible inter-regional directed relationship, to characterize aging effects on structural associations between core brain regions within each of these structural sensory/motor networks using volumetric MRI data. We then further examined the discriminability of BN models for the young (*N* = 109; mean age =22.73 years, range 20–28) and old (*N* = 82; mean age =74.37 years, range 60–90) groups. The results of the BN modeling demonstrated that structural associations exist between two homotopic brain regions from the left and right hemispheres in each of the three networks. In particular, compared with the young group, the old group had significant connection reductions in each of the three networks and lesser connection numbers in the visual network. Moreover, it was found that the aging-related BN models could distinguish the young and old individuals with 90.05, 73.82, and 88.48% accuracy for the auditory, visual, and motor networks, respectively. Our findings suggest that BN models can be used to investigate the normal aging process with reliable statistical power. Moreover, these differences in structural inter-regional interactions may help elucidate the neuronal mechanism of anatomical changes in normal aging.

## Introduction

Structural magnetic resonance imaging (MRI) studies have demonstrated aging-related anatomical alterations in the brain. As we age in general, the global gray and white matter volumes decrease and the volume containing cerebrospinal fluid increases (Good et al., 2001; Sowell et al., 2003; Lemaitre et al., 2012). Moreover, structural changes are heterogeneous across different brain regions in normal aging. The prominent aging effects were observed mainly in the frontal cortex and some parts of the temporal lobe (Fjell et al., 2009; Peelle et al., 2012; Ziegler et al., 2012; Fjell et al., 2013). Reports on these findings primarily interested in isolated regional/global brain structural changes utilizing univariate approaches, such as voxel-based morphometry (VBM) or regions of interest (ROIs). These studies laid important foundation to localize aging-associated brain areas or to investigate brain structural trajectories in the life span. Based on the knowledge gained from these previous works, it becomes possible to understand the aging-related changes by seeking out the covariance information of morphological features across various brain regions over its whole volume.

Multivariate analytical methods, which aim to determine the interrelationship among brain regions, have been extensively applied to neuroimaging studies to explore the brain systems in different population. Based on the existing literature, it is well-known that the human brain is intrinsically organized into complex functional and structural networks (Fox et al., 2005; Fox and Raichle, 2007; Bullmore and Sporns, 2009; Bassett and Gazzaniga, 2011). Recent multivariate MRI studies have revealed aging-related alterations in not only functional but also structural networks. For example, in addition to the resting-state functional magnetic resonance imaging (rs-fMRI) studies documenting age-related changes in functional connectivity in resting-state functional networks, such as the default-mode network (DMN), motor and visual networks (Ferreira and Busatto, 2013), several structural MRI studies have reported that age-associated regional covariance networks involve widespread areas of the cortex and subcortex (Alexander et al., 2006; Brickman et al., 2007; Bergfield et al., 2010). Reduced structural associations were also observed in the structural covariance networks subserving the language-related semantic network, the executive control network, and the DMN in older subjects compared with younger subjects (Montembeault et al., 2012). In addition, a graph theoretical analysis of brain structural networks demonstrated that the topological patterns exhibit a modular organization and that the aging brain exhibits reduced intra-/inter-modular connectivity in modules corresponding to the executive function and the DMN, resulting in the development of a more localized network organization in old age (Chen et al., 2011; Wu et al., 2012; Zhu et al., 2012). In contrast to the investigation of the effects of aging on the networks related to high-level cognitive functions, however, reports of the same nature but on the low-level sensory/motor systems, especially viewed as networks, are rare.

Bayesian networks (BN), as a powerful multivariate technique, enables investigation of specific conditional probability dependencies over a set of random variables, or the nodes in BN. In applying to the neuroimaging studies, these nodes are usually the predefined set of ROIs. The measurements obtained from each of the ROIs, either fMRI time-series or structural MRI data, are viewed as realizations of the corresponding random variable. At present, BN has been widely and successfully utilized to investigate brain connectivity relationships for both functional and structural MRI studies (Chen and Herskovits, 2006; Zheng and Rajapakse, 2006; Wu et al., 2011; Chen et al., 2012; Wang et al., 2014) and other studies (Bielza et al., 2011, 2013; Larrañaga et al., 2013). For fMRI data, BN modeling examines conditional dependencies of brain activity among the sets of the given brain regions for each individual subject. For structural MRI data, BN modeling investigates probabilistic associations of morphological feature based on morphometric estimations such as the average of gray matter volume of brain regions over multi-subjects at the group level either within or between groups. Our previous study examined the aging influence on the gray matter structural inter-regional linkage within the DMN utilizing BN modeling. Our findings showed that the older subjects exhibited significant structural association alterations among core brain regions within the DMN compared to their younger counterparts and indicated that the BN modeling approach can potentially serve as a useful tool for studying structural probabilistic dependence among multiple brain regions (Wang et al., 2014).

Overall, the DMN, visual, auditory, and motor networks are the four most common brain networks. However, to date, the majority of the aging-related studies focused on the DMN. The auditory, visual, and motor networks have received much less attention in normal aging research. The anatomical inter-regional relationships among distinct brain areas may potentially hold important implications on structural networks of the aging brain. In this study, we employed Gaussian BN to characterize the aging-related structural association alterations between core brain regions within each of these three structural networks from structural MRI data and further investigated the discriminability of the aging-associated BN models for the young and old groups.

## Materials and methods

### Participants

The data originated from a large public database of the Open Access Series of Imaging Studies (OASIS) (http://www.oasis-brains.org). There were 191 healthy right-handed subjects, including 109 younger (65 females and 44 males, mean ± *SD* age = 22.73 ± 2.34, range 20–28) and 82 older individuals (60 females and 22 males, mean ± *SD* age = 74. 37 ± 8.23, range 60–90). These two groups did not significantly differ in their sex ratio [χ^2^_(1)_ = 3.79, *p* > 0.05]. Younger healthy subjects were questioned before image acquisition by a trained technician about their medical histories and use of psychoactive drugs (Marcus et al., 2007). Older healthy adults underwent the ADRC's (Washington University Alzheimer Disease Research Center) full clinical assessment, and their status of dementia was determined using the Mini-Mental State Examination (MMSE) and Clinical Dementia Rating (CDR). Only normal older subjects (CDR = 0; mean MMSE score = 29.02 ± 1.27) were included in our study. All subjects participated in accordance with guidelines of the Washington University Human Studies Committee. The detailed demographic information on the participants was described by (Marcus et al., 2007).

### Data acquisition

sMRI scanning was performed on a 1.5 Tesla Vision scanner (Siemens, Erlangen, Germany). For each participant, three to four T1-weighted, sagittally oriented 3D anatomical images were acquired using a magnetization prepared rapid acquisition gradient-echo (MPRAGE) sequence (*TR* = 9.7 ms, *TE* = 4.0 ms, *TI* = 20 ms, flip angle = 10°, field of view = 256 × 256 mm, voxel size = 1 × 1 mm, slices = 128 and thickness = 1.25 mm) in a single imaging session. For the sake of high signal-to-noise, the T1 image selected in this study for each subject was an average image of all motion-corrected and coregistered images (Marcus et al., 2007).

### Preprocessing

The spatial preprocessing of sMRI data was performed with a VBM protocol (Ashburner and Friston, 2000; Good et al., 2001; Ashburner, 2007) in SPM8 (Statistical Parametric Mapping, http://www.fil.ion.ucl.ac.uk/spm) using the VBM8 Toolbox (http://dbm.neuro.uni-jena.de/vbm8). The VBM8 procedure involved two major steps: segmentation and normalization. The VBM8 segmentation approach applies an adaptive maximum a posteriori (MAP) (Rajapakse et al., 1997) and a partial volume estimation (PVE) (Tohka et al., 2004) to segment the native space images into rigid-body aligned gray and white matter images in the Montreal Neurological Institute (MNI) space for all subjects. Subsequently, two denoising methods, a spatially adaptive non-local means (SANLM) denoising filter (Manjon et al., 2010) and a classical Markov Random Field (MRF) approach, were utilized to improve the segmentation (Rajapakse et al., 1997). A high-dimensional normalization protocol, referred to as diffeomorphic anatomical registration using exponentiated Lie algebra (DARTEL), was employed (Ashburner, 2007; Ashburner and Friston, 2011). DARTEL utilizes the large-deformation parameterized by a single constant velocity field to generate diffeomorphic and invertible deformations. The initial tissue probability templates were created by averaging the rigid-body aligned tissue segments for each subject. The image normalization was then based on the created templates. Image registration and template creation were iteratively implemented to obtain the individual deformation fields. At the end of the iteration, the gray matter maps were deformed to their own increasingly crisp average templates and further normalized to the Montreal Neurological Institute (MNI) space. Thereafter, the normalized gray matter partitions were multiplied by the Jacobian determinants from the deformations to preserve the total amount of tissue in the native spaces. The voxel intensity value indicates the absolute amount (volume) of gray matter in the modulated gray matter images (Good et al., 2001). Zero means that there is no gray matter in that voxel, and larger voxel intensity value means more amount of gray matter. Finally, the gray matter maps for all subjects were smoothed with an 8-mm full width at half maximum (FWHM) Gaussian kernel. After the above preprocessing, we obtained the smoothed, modulated, and normalized gray matter volume images from both groups.

### ROIs definition

Based on the findings from the existing rs-fMRI (Damoiseaux et al., 2006; De Luca et al., 2006; Mantini et al., 2007) and structural covariance networks studies (Zielinski et al., 2010; Montembeault et al., 2012), we defined core ROIs for the auditory, visual and motor networks, respectively. Each ROI mask was generated using the WFU_PickAtlas software (http://www.ansir.wfubmc.edu) (Maldjian et al., 2003). Table 1 shows the brain region names, corresponding abbreviations, and AAL labels of these ROIs for each network.

**Table 1:** **Core ROIs for the auditory, visual and motor networks**.

**Brain regions**	**Abbr**.	**AAL labels**
**AUDITORY NETWORK**		
Left heschl gyrus	lHES	Heschl_L
Right heschl gyrus	rHES	Heschl_R
Left supramarginal gyrus	lSMG	SupraMarginal_L
Right supramarginal gyrus	rSMG	SupraMarginal_R
Left superior temporal gyrus	lSTG	Temporal_Sup_L
Right superior temporal gyrus	rSTG	Temporal_Sup_R
**VISUAL NETWORK**		
Left calcarine cortex	lCAL	Calcarine_L
Right calcarine cortex	rCAL	Calcarine _R
Left lingual gyrus	lLING	Lingual_L
Right lingual gyrus	rLING	Lingual_R
Left middle occipital gyrus	lMOG	Occipital_Mid _L
Right middle occipital gyrus	rMOG	Occipital_Mid_R
**MOTOR NETWORK**		
Left postcentral gyrus	lPoCG	Postcentral_L
Right postcentral gyrus	rPoCG	Postcentral_R
Left Precentral gyrus	lPreCG	PreCentral_L
Right Precentral gyrus	rPreCG	PreCentral_R
Supplementary motor area	SMA	Supp_Motor_Area

Every ROI covered the entire area of the corresponding anatomical region defined by the AAL atlas. The average gray matter volumes of each ROI in the modulated gray matter images were calculated after thresholding all voxel intensities above a 0.15 cut-off value to exclude the possible non-gray matter voxels within the ROI. For each of the three networks, the average gray matter volumes of ROIs from each subject were used as the set of continuous variables to construct Gaussian BN models for the young and old groups.

### Bayesian network modeling

A BN consists of a graphical model *G* and a set of parameters θ. The graphical model *G* is a directed acyclic graph (DAG) composed of nodes (random variables) and directed edges encoding conditional independence relationships about the variables. The parameters θ denote the collection of parameters of the conditional probability distributions over node variables. BN is an approach to decompose the joint probability distribution of the given set of random variables as the product of most economic conditional probability distributions (Zheng and Rajapakse, 2006; Mumford and Ramsey, 2014). With the assertion of conditional dependencies and independencies encoded by DAG, the joint probability density for all nodes can be described as the following formula:

(1) $$p(X_{1},X_{2},\cdots ,X_{n})=\prod_{j\;=\;1}^{n}p(X_{j}|\pi_{j})$$

where *n* is the number of nodes, the conditional probability density *p*(*X_j_*|π_*j*_) can be calculated for node *j* given its parent node-set π_*j*_, which directly connects with node *j* in the graph structure *G*. In the case the variables are normally distributed (Gaussian variables), we have:

(2) $$p(X_{j}|\pi_{j})=\frac{1}{\sqrt{2\pi }\sigma_{j}}\exp\left[-\frac{1}{2\sigma_{j}^{2}}{\left(X_{j}-\mu_{j}\right)}^{2}\right]$$

where μ_*j*_ = *u_j_* + ∑_*X_p_* ∈ π_*j*__ β_*p*_(*X_p_* − *u_p_*), μ_*j*_, and σ_*j*_ represent the conditional mean and variance of *X*_*j*_ given its parent node-set π_*j*_, respectively. β_*p*_ denote connection weight coefficients from parent node variables *X*_*p*_ to node *X*_*j*_; In addition, *u*_*j*_ and *u*_*p*_ represent the unconditional mean of *X*_*j*_ and *X*_*p*_, respectively. In this study, *X*_*j*_ represents the average gray matter volume of the pre-specified ROI *j*; *n* is the number of ROIs for each network.

In a BN, the probabilistic relationship is in context of conditional independencies, that is, a variable is conditionally independent of the rest variables of the DAG given its parents. Then if there is no edge between two variables, which implies that they are independent conditioned on the rest of the variables. However, if there is the directed edge from node *X*_1_ (parent node) to node (child node) *X*_2_, which represents the probabilistic dependence of *X*_2_ on *X*_1_ conditioned on all other variables in the DAG. In this study, the probabilistic dependence, representing the interdependent volume relationship among these brain regions, is phrased as a “direction” from one brain region to another, as in other MRI studies using BN modeling (Chen and Herskovits, 2006; Wu et al., 2011; Chen et al., 2012). In another words, the directionality represents the association dependency between gray matter volumes of brain regions in the context of conditional probability in this study.

The BN modeling was implemented with the Bayesian Net Toolbox (https://code.google.com/p/bnt/) and the DAGLearn Toolbox (http://www.cs.ubc.ca/~murphyk/Software/DAGlearn/index.html) in MATLAB R2010. In the present study, the BN model was constructed from gray matter volume data by means of the search-and-score approach and the maximum likelihood estimation (MLE) algorithm to determine the DAG structure configuration together with the corresponding parameters. The search-and-score approach used the greedy search algorithm (Chickering, 2003) and the Bayesian Information Criterion (BIC) score to obtain an optimal BN model. The greedy search algorithm searches and assesses the possible DAGs by adding, removing, and reversing edges between any two nodes until it returns the one with the highest BIC score. The BIC cost function is described as the following formula:

(3) $$BIC(\theta )=\log p(D|\hat{\theta })-\frac{d}{2}\log n$$

where log *p* (*D*|$\hat{\theta }$) is the maximized log-likelihood of data *D* conditional on $\hat{\theta }$, indicating the fitness degree of the model to the data; $\hat{\theta }$ is the MLE of the parameters; the second term $\frac{d}{2}$ log *n* in the BIC formula above is the penalty on the model complexity; *d* is the number of free parameters of the model.

As previously stated, we assumed in this study that data follows a Gaussian distribution. Under this assumption, the linear Gaussian BN can be represented as a set of multivariate linear relationships (Zheng and Rajapakse, 2006; Wu et al., 2011). That is, for each node *X*_*j*_ in the Gaussian BN, it can be considered as the linear equation of its parent nodes π_*j*_ in this network, and the DAG edges were assigned the regression coefficients as connection weight coefficients from parent node-set π_*j*_ to node *X*_*j*_. The weight coefficient indicates the strength of the structural association or gray matter volumetric correlation among different brain regions in this study (Chen and Herskovits, 2006).

After BN learning, for each of the three networks, a unique BN model was derived for each group. Thus, we obtained 3 optimal BN model pairs for the young and old groups: the auditory, visual, and motor networks pairs, respectively. For each BN model pair, the between young/old -group differences in all connection weight coefficients were assessed by a non-parametric permutation test. Here, the connection is about the existence of an directed edge between two brain regions in the BN models and represents a conditional probabilistic dependence between the corresponding variables. For a given “directed” edge in the original BN (without the subjects' group membership random permutation), the permutation test is to examine the random chance of the given direction with the given connection strength (the type-I error). The difference in connection weight coefficients between two groups was calculated as the test statistic (the real group difference). The subject-group membership was randomly assigned for each subject to form two new groups (that is, to permute the subject labels randomly). Then a BN model was constructed for each new group and the difference of the connection weight coefficients between these two groups were calculated as the permuted group statistic. Finally, in total 5000 permutations, the proportion of the permuted group statistic value less than the test statistic value was calculated as the estimated probability for each connection weight coefficient. This process was repeated 5000 times, for the auditory, visual, and motor networks respectively. The significance level was controlled by the type-I error probability of Young>Old or Old>Young at *p*-value of 0.05.

### Assessing discriminability of the BN models

After constructing the BN model, we used the conditional probability densities resulted from the learned structure and parameters to form the joint density for the young and old groups separately. Then we utilized the joint probability density function to compute the likelihood of a subject belonging to one group or the other. Given ROIs data from a subject, we could assign the group membership of this given subject by comparing the joint probability density values between two BN models. Subsequently, the receiver operating curve (ROC) analysis is implemented to assess the discriminability of the BN models for the auditory, visual, and motor networks respectively. The classification accuracy was defined as N_Y_/(N_Y_+N_N_), in which N_Y_ was the number of subjects that were correctly identified and N_N_ was the number of subjects that were not correctly identified.

## Results

### Structural associations within each of the three networks

**Figure 1:**
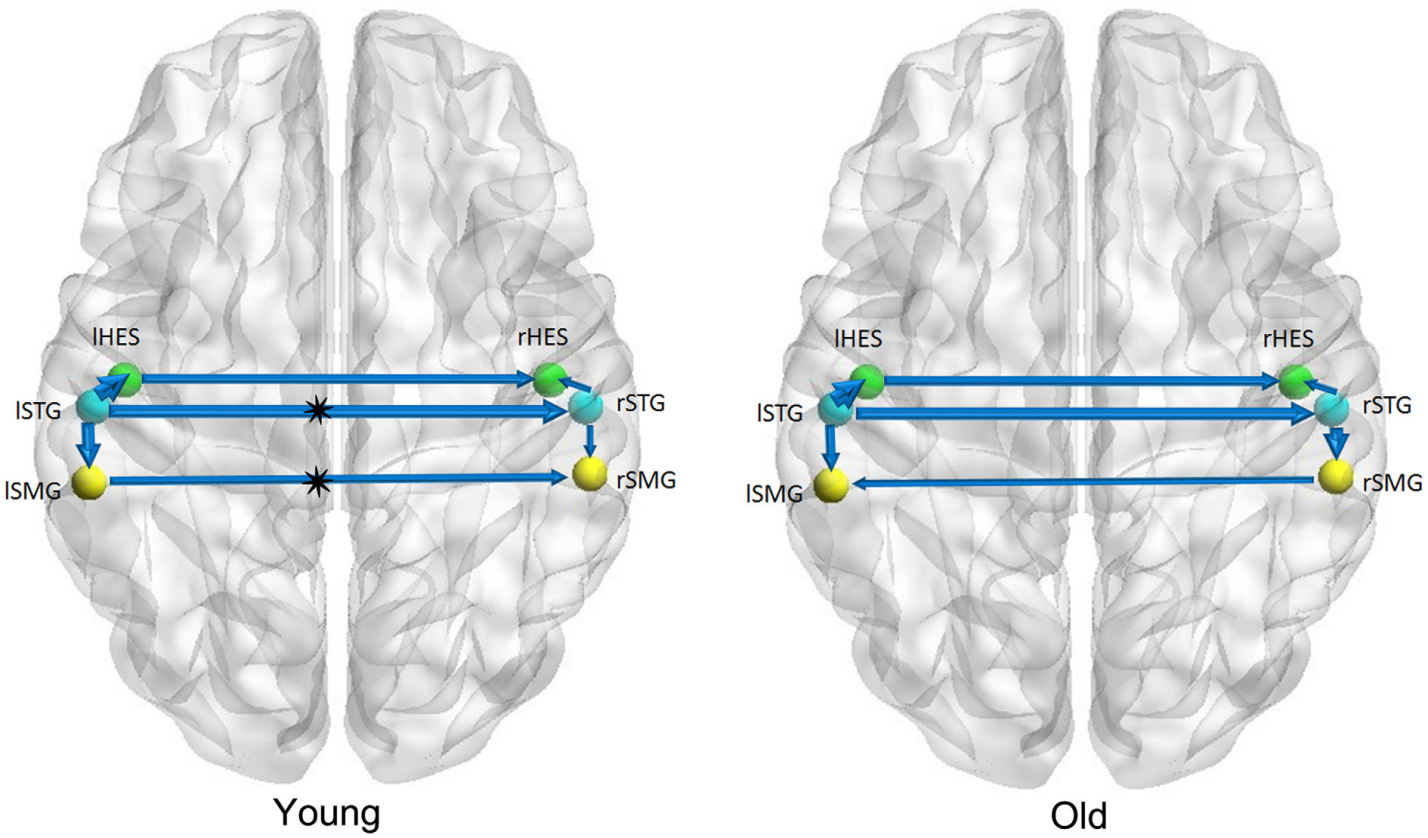
**Bayesian network model of the auditory network in the Young (left panel) and Old (right panel) groups**.

**Figure 2:**
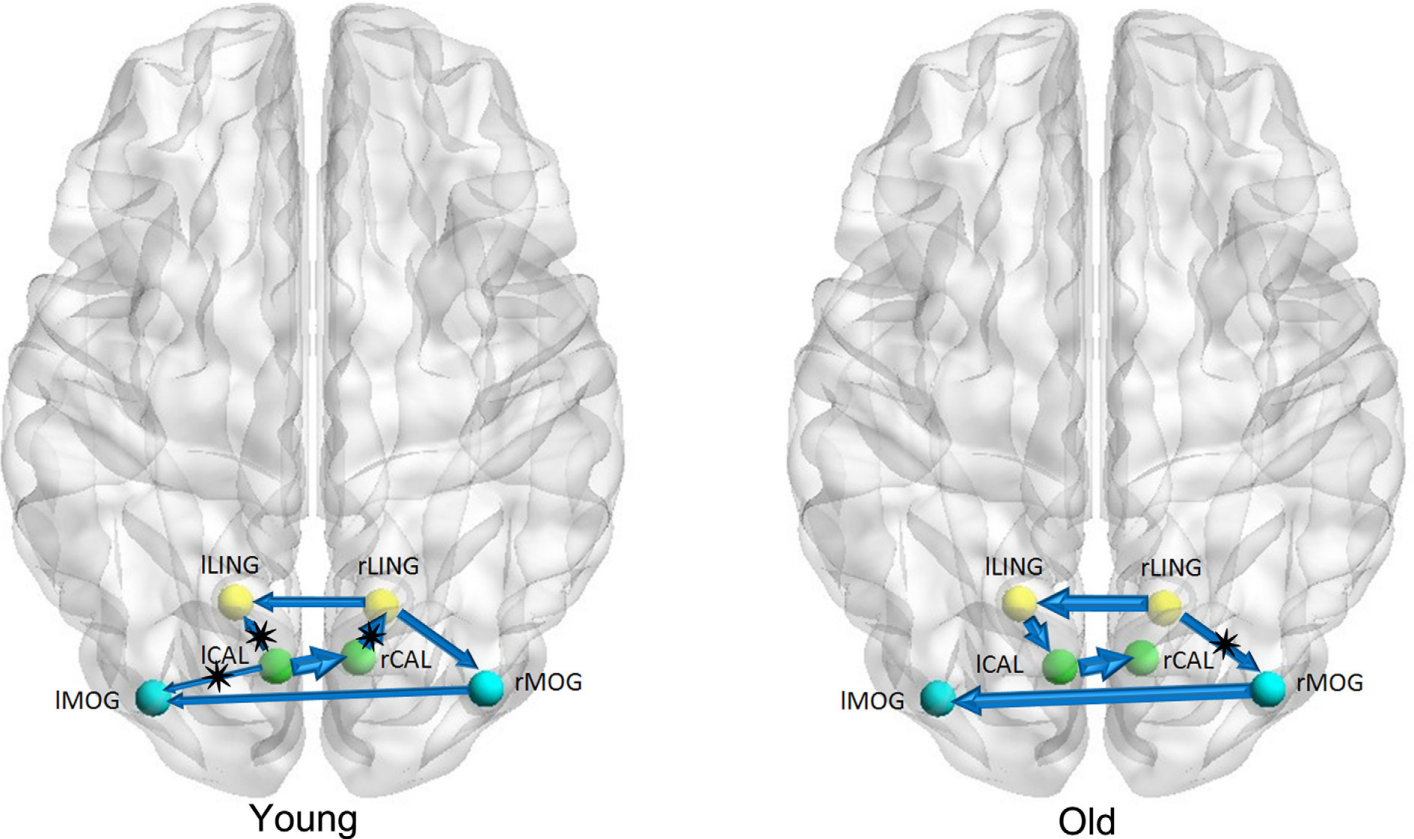
**Bayesian network model of the visual network in the Young (left panel) and Old (right panel) groups**.

**Figure 3:**
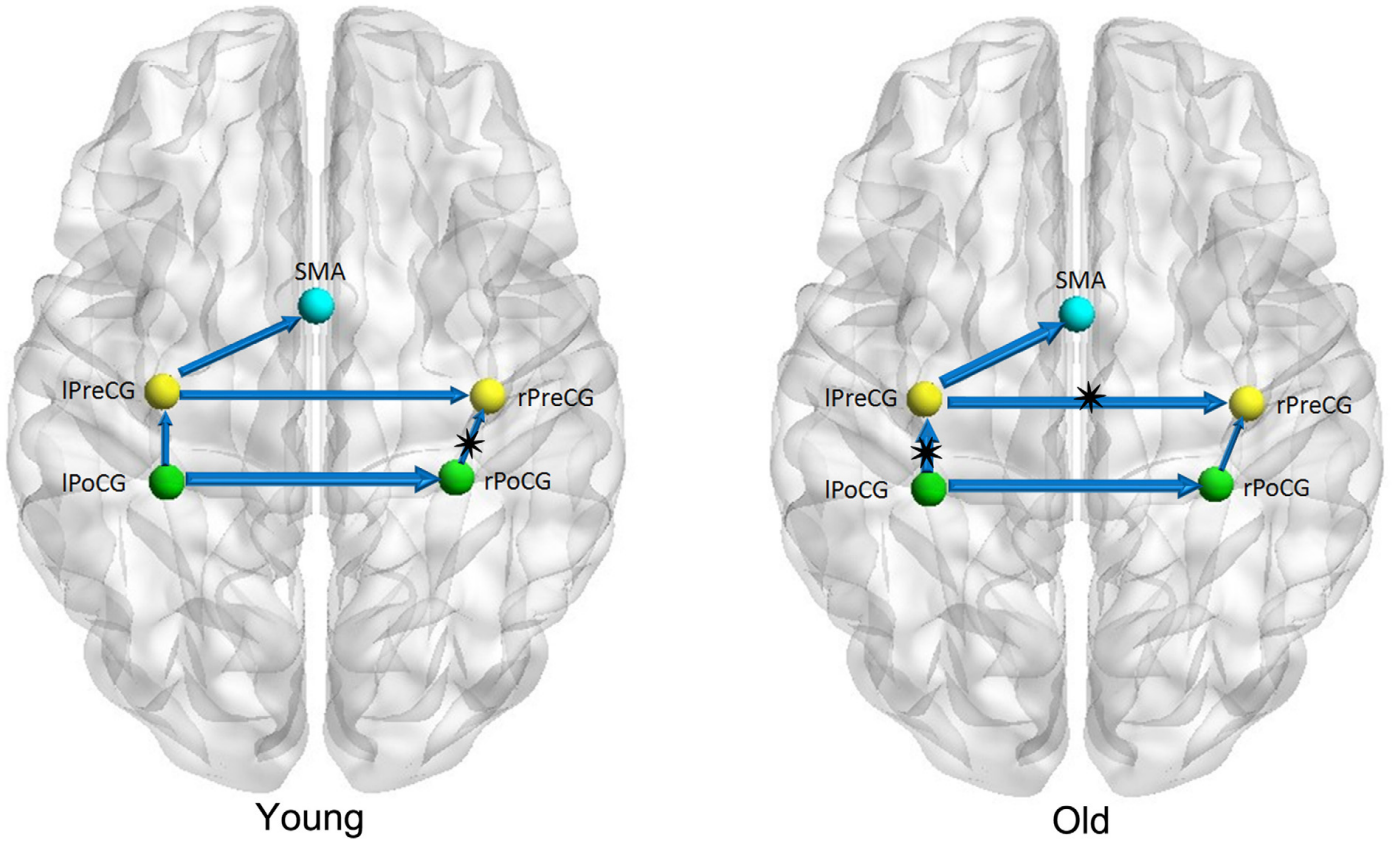
**Bayesian network model of the motor network in the Young (left panel) and Old (right panel) groups**.

Figures 1–3 show two BN models of the young and old groups for the auditory (Figure 1), visual (Figure 2) and motor networks (Figure 3), respectively. In Figures 1–3, the arrows represent dependencies among brain regions, and the thickness of the arrows is proportional to the strength of the connections. The asterisks indicate the connections that are significantly stronger in the Young/Old than in Old/Young groups. Table 2 lists the corresponding connection directions and weight coefficients.

**Table 2:** **List of connections and weight coefficients in the Bayesian network models of the young and old groups**.

**Connections**		**Weight coefficients**	
		**Young**	**Old**
**AUDITORY NETWORK**			
I	lHES→rHES	0.502	0.493
	lSTG→rSTG	0.665	0.634
	lSTG→lHES	0.786	0.811
	rSTG→rHES	0.328	0.346
	lSTG→lSMG	0.654	0.494
	rSTG→rSMG	0.338	0.691
III	lSMG→rSMG	0.443	
	rSMG→lSMG		0.351
**VISUAL NETWORK**			
I	rLING→lLING	0.533	0.841
	rMOG→lMOG	0.438	0.786
	lCAL→rCAL	0.897	0.914
	rLING→rMOG	0.537	0.563
II	rCAL→rLING	0.758	
	lCAL→lMOG	0.321	
III	lCAL→lLING	0.369	
	lLING→lCAL		0.714
**MOTOR NETWORK**			
I	lPreCG→rPreCG	0.483	0.621
	lPoCG→rPoCG	0.698	0.657
	lPoCG→lPreCG	0.396	0.633
	rPoCG→rPreCG	0.344	0.326
	lPreCG→SMA	0.487	0.671

*Part I of this table lists connections in both the young and old groups. Part II lists the connections present only in the young group. Part III lists the connections with opposing direction in the two groups*.

### Between-group association differences

Table 3 lists the probabilities of type-I errors regarding the between-group differences in the connection weight coefficients. In Table 3, the column “Young>Old” displays the type-I errors probabilities of the connections in the young group are stronger than in those in the old group. The other column, “Old>Young,” shows the opposite.

**Table 3:** **Type-I error probabilities of the between-group connection differences**.

**Young>Old**		**Old>Young**	
**Connections**	**Probability**	**Connections**	**Probability**
**AUDITORY NETWORK**			
lHES→rHES	0.400	lHES→rHES	0.600
lSTG→rSTG	**0.000**	lSTG→rSTG	1.000
lSTG→lHES	0.619	lSTG→lHES	0.381
rSTG→rHES	0.920	rSTG→rHES	0.080
lSTG→lSMG	0.287	lSTG→lSMG	0.713
rSTG→rSMG	0.912	rSTG→rSMG	0.088
lSMG→rSMG	**0.050**	rSMG→lSMG	0.231
**VISUAL NETWORK**			
rLING→lLING	0.761	rLING→lLING	0.239
rMOG→lMOG	0.874	rMOG→lMOG	0.126
lCAL→rCAL	0.757	lCAL→rCAL	0.243
rLING→rMOG	0.952	rLING→rMOG	**0.048**
rCAL→rLING	**0.000**	lLING→lCAL	0.183
lCAL→lMOG	**0.000**		
lCAL→lLING	**0.002**		
**MOTOR NETWORK**			
lPreCG→rPreCG	0.982	lPreCG→rPreCG	**0.018**
lPoCG→rPoCG	0.394	lPoCG→rPoCG	0.606
lPoCG→lPreCG	0.977	lPoCG→lPreCG	**0.023**
rPoCG→rPreCG	**0.004**	rPoCG→rPreCG	0.996
lPreCG→SMA	0.918	lPreCG→SMA	0.082

*The probabilities marked in bold indicate significantly stronger connections (p < 0.05)*.

For the auditory network, the old subjects had significant connection reductions from the left superior temporal gyrus (STG) to the right STG and from the left supramarginal gyrus (SMG) to the right SMG compared with the young subjects. For the visual network, the old subjects had significant connection reductions from the right calcarine cortex (CAL) to the right lingual gyrus (LING), from the left CAL to the left middle occipital gyrus (MOG) and from the left CAL to the left LING and connection increases from the right LING to the right MOG. For the motor network, significant connection reductions in the old subjects were found from the right postcentral gyrus (PoCG) to the right precentral gyrus (PreCG), and connection increases from the left PreCG to the right PreCG and from the left PoCG to the left PreCG were also observed.

### Discriminability of the BN models

Based on the derived BN models, the joint probability density values for the auditory, visual and motor networks distinguished the older adults from the younger adults with 89.02, 75.61, and 85.37% sensitivity, respectively, 90.83, 72.48, and 90.83% specificity, respectively, and 90.05, 73.82, and 88.48% accuracy, respectively.

## Discussion

In this study, we constructed the BN models of the auditory, visual, and motor networks based on regional gray matter volumetric data from the old and young groups. We then examined the between-group differences in connection with weighted coefficients by a nonparametric permutation test. The results of the BN modeling showed that, for both young and old groups, there were 7 connections in the auditory network and 5 connections in the motor network, but 5 connections for the old group and 7 connections for the young group in the visual network. We further examined the discriminability of the aging-associated BN models for each network.

The results of the BN modeling demonstrated that the structural associations exist between two homotopic brain regions from the left and right hemispheres in each of the three networks, such as the bilateral heschl gyri (HES), STG and SMG in the auditory network, bilateral LING, MOG, and CAL in the visual network, and bilateral PreCG and PoCG in the motor network (Figures 1–3 and Table 2). Some previous structural MRI studies indicated that the morphology of the human brain covaried in gray matter density/volume or cortical thickness; moreover, such structural covariance would change owing to different clinical conditions (Mechelli et al., 2005; Andrews-Hanna et al., 2007; Evans et al., 2008; Seeley et al., 2009; Zielinski et al., 2010). Mechelli et al. reported structural correlations in the human cortex and observed homotopic effects in the inferior frontal, primary auditory and somatosensory cortex that are consistent with our findings, although with the exception of primary visual cortex (Mechelli et al., 2005). The existing diffusion tensor imaging (DTI) studies reconstructed and visualized prominent white matter tracts in the human brain (Wakana et al., 2004; van den Heuvel et al., 2009). Van den Heuvel et al. reconstructed the fiber tract pathways between the functionally linked brain areas of several resting-state networks and demonstrated the existence of direct white matter connections in these resting-state networks, such as the well-known DMN, primary motor and visual networks (van den Heuvel et al., 2009). The above MRI studies support our findings of structural associations between two homotopic regions from two hemispheres in each of the three networks.

We found that there exist 7 connections in the auditory network for both the old and young groups. Compared with the young subjects, the old subjects had significant connection reductions from the left STG to the right STG and from the left SMG to the right SMG. Using a cortical thickness and graph theory analysis, Chen et al. investigated age-related alterations in the modular structure networks and found a reducing trend for the intra-modular connectivity in the auditory module in the aging group (Chen et al., 2011), which is in agreement with our findings. Hearing loss is one of the major impairments in the older population and causes difficulties in speech comprehension and communication (Peelle et al., 2011; Eckert et al., 2012). Lower gray matter volumes were observed in the primary auditory cortex (Husain et al., 2011; Peelle et al., 2011), moreover, Peelle et al. demonstrated a significant linear relationship between the hearing ability and the gray matter volume in primary auditory regions. Hence, such findings support the view that age-related hearing loss is associated with neuroanatomical alterations in the auditory cortex.

For the visual network, there were 5 connections for the old group but 7 connections for the young group. The old subjects had significant connection reductions from the right CAL to the right LING, from the left CAL to the left MOG and from the left CAL to the left LING and connection increases from the right LING to the right MOG. Based on the regional gray matter volume, Wu et al. applied graph theory to investigate age-related changes in the topological organization of structural brain networks and depicted that the old group showed a prominent decrease in the connector ratio and the intermodular connections in comparison to the young and middle groups; thus, the modular organizations associated with memory, auditory and visual systems in the old group are quite different from those in the younger groups (Wu et al., 2012), in agreement with our findings. In addition, Wu et al. also characterized the age-related longitudinal changes in the structural brain networks and found significant negative correlations between the baseline age and the annual rate of change in nodal strength in the brain regions primarily involved in the visual and motor systems (Wu et al., 2013). Van den Heuvel et al. found that tracts of the splenium of the corpus callosum connected the bilateral brain regions of the primary visual network (van den Heuvel et al., 2009), and Ciccarelli et al. demarcated the optic radiations as the major fiber tract in the visual network (Ciccarelli et al., 2003). The existence of these white matter tracts provides the subserving foundation for structural associations in gray matter volume.

Five connections were present in the motor network for both groups. In the old subjects, significant connection reductions were found from the right PoCG to the right PreCG compared to the young subjects, and connection increases from the left PreCG to the right PreCG and from the left PoCG to the left PreCG were also found. Older adults exhibit motor performance deficits with advanced aging, and these deficits are accompanied by age-related atrophy of the motor cortical regions and corpus callosum and biochemical changes; moreover, larger gray matter volume and better white matter integrity are correlated with better motor performance for review see Seidler et al. (2010). Some studies have indeed depicted accelerated loss of gray matter volume in the local sensorimotor cortex, including the SMA, and the bilateral precentral and postcentral gyri in older adults (Good et al., 2001; Resnick et al., 2003; Kalpouzos et al., 2009). Van den Heuvel et al. found that tracts of the body of the corpus callosum connected the primary sensorimotor network (van den Heuvel et al., 2009), and some other DTI studies demonstrated the degenerative changes in the corpus callosum in normal aging (Kochunov et al., 2009; Sala et al., 2012). Using the cortical thickness as a morphometric measurement in structural MRI, Chen et al. found significant correlation strength increases among the bilateral frontal regions involved with the bilateral precentral gyri in older adults in comparison to their younger counterparts (Chen et al., 2011), which is consistent with our findings.

In this study, the BN model depicts the conditional probability dependencies based on the average of gray matter volume of core brain regions within each of the three networks, and the differences in such conditional dependencies exhibit the aging effects at a global level. Once the BN model is constructed, it can be used as an effective tool for classification. Hence, we used the derived BN models to identify the membership between the older and younger groups. The results showed that the auditory network had the highest statistical discriminability with 89.02% sensitivity, 90.83% specificity and 90.05% accuracy. The sensitivity, specificity, and accuracy were relatively low for the visual network, but all three were above 70%. Overall, our findings suggest that the aging-related BN models could predict the group distinction with reliable statistical power.

It should be noted that the changes in gray matter volume of the brain regions within these three networks have not always been consistent from the existing structural MRI studies. Some publications in the literature reported these brain regions to be preserved with aging, whereas others have indeed reported gray matter atrophy in most of these brain regions. This discrepancy may result from the different methods applied or the samples selected in these studies. In the present study, we applied a multivariate BN approach and focused on the probabilistic associations over a set of gray matter volume estimations in a specific network but not the single brain region alterations (e.g., volume increases or decreases). A number of publications have suggested that structural covariances or associations between functionally connected brain regions may result from mutually trophic influences by white matter fiber connections or common experience-related plasticity (Ferrer et al., 1995; Mechelli et al., 2005; Soriano-Mas et al., 2013). The alterations of dependence between brain regions may arise from a lack of mutually trophic influences in different clinical conditions (He et al., 2008; Seeley et al., 2009).

Most of the previous MRI studies have documented structural association decreases in the structural networks that promote high-order cognitive functions, such as the language-related semantic network, the executive control network and the DMN, in older adults compared with younger adults (Chen et al., 2011; Montembeault et al., 2012; Wu et al., 2012). Our previous study also examined the aging influence on gray matter structural associations within the DMN utilizing BN (Wang et al., 2014). As we all know, the auditory, visual, and motor function is related to basic abilities in life, and these impairments will lead to a diminished quality of life in the older. Although our present study does not provide the direct biological evidence of such structural inter-regional associations, we expect these preliminary findings of age-related alterations to help elucidate the anatomical changes in these three networks.

About BN methodology, BN can be learned in an unsupervised manner from data without prior knowledge. By compactly representing the joint probability distribution over a set of random variables, BN model can be used to examine probabilistic associations among these variables in an exploratory manner. Till now, BN approach has been successfully utilized in brain neuroimaging studies. BN modeling, as a valuable method of mining association relationships between continuous variables, can be used to investigate the association dependency based on regional gray matter volumes. In our study, the directed edge representing a conditional probabilistic dependence cannot be viewed, without any direct biological and medical evidence, as the biological causal relationship (Chen and Herskovits, 2006). In addition, the BN model constructed can be used as an effective tool for discriminating the group membership. We hope that our study provided additional, but preliminary or exploratory findings in the existing investigations.

In conclusion, we characterized the influence of aging on interregional gray matter structural associations within each of the auditory, visual, and motor networks. Our findings suggest that the BN model can detect between-group differences with reliable statistical power. These differences in structural associations may further provide new insights into the neuronal mechanisms of morphometric changes in normal aging.

## Author contributions

Designed the work: Xiaojuan Guo, Kewei Chen, and Li Yao. Analyzed the data: Yan Wang, Xia Wu, and Jiacai Zhang. Interpreted the data: Xiaojuan Guo, Ke Li, Zhen Jin, and Li Yao. Wrote the paper: Xiaojuan Guo and Kewei Chen. Revised the paper: Xiaojuan Guo, Kewei Chen, and Li Yao.

### Conflict of interest statement

The authors declare that the research was conducted in the absence of any commercial or financial relationships that could be construed as a potential conflict of interest.
